# TNK2 preserves epidermal growth factor receptor expression on the cell surface and enhances migration and invasion of human breast cancer cells

**DOI:** 10.1186/bcr2087

**Published:** 2008-04-24

**Authors:** Jillian Howlin, Jeanette Rosenkvist, Tommy Andersson

**Affiliations:** 1 Cell and Experimental Pathology, Lund University, Department of Laboratory Medicine, Clinical Research Centre, Ent 72, Bldg 91, fl 11, Malmö University Hospital, S-205 02 Malmö, Sweden

## Abstract

**Introduction:**

Amplification of the TNK2 gene in primary tumours correlates with poor prognosis. In accordance, TNK2 overexpression was shown to promote invasion of cancer cells – but the mechanism by which TNK2 mediates these effects is unresolved. TNK2 was suggested to regulate Cdc42-driven migration by activation of breast cancer antioestrogen resistance 1 (BCAR1); however, distinct from this effect is evidence for a role of TNK2 in the regulation of epidermal growth factor receptor (EGFR) endocytosis and degradation. In the present study we sought to investigate whether negative targeting of TNK2 by siRNA could be used to inhibit cancer cell invasion, to establish the contribution of its effect on the EGFR and to consequently attempt to resolve the issue of TNK2's mechanism of action.

**Methods:**

We used siRNA to knockdown expression of TNK2 and its proposed effector BCAR1 in order to analyse the effect of this knockdown on cancer cell behaviour *in vitro*. We examined morphological changes using phase-contrast microscopy and immunohistochemistry. Functional parameters examined included apoptosis, proliferation, migration and invasion. We also performed flow cytometry analysis to examine EGFR cell surface expression and carried out western blot to examine the total EGFR levels.

**Results:**

We observed that targeting of TNK2 by siRNA in breast cancer cells resulted in distinct morphological changes characterised by a stellate appearance and an absence of protrusions at membrane edges. These changes were not recapitulated upon siRNA targeting of BCAR1. We thus hypothesised that a component of the effects induced by TNK2 may be independent of BCAR1. Consistent with the idea of an alternative mechanism for TNK2, we observed that TNK2 associates with activated EGFR in breast cancer cells in a TNK2-kinase-independent manner. Furthermore, we demonstrated that TNK2 functions to maintain EGFRs on the cell surface. We could demonstrate that the main functional effect of activating these surface EGFRs in breast cancer cells is stimulation of migration. In accordance, TNK2 silencing by siRNA led to a significant reduction in cell surface EGFR and to a concomitant decrease in the migratory and invasive capacity of breast cancer cells.

**Conclusion:**

Our data suggest that TNK2 can enhance migration and invasion of breast cancer cells via preservation of EGFR expression, notwithstanding its previously reported signalling via BCAR1, explaining its oncogenic behaviour *in vitro *and correlation with metastatic human breast cancer *in vivo*.

## Introduction

The human epidermal growth factor receptor (EGFR) is overexpressed in up to 20% of patients diagnosed with breast cancer and is associated with reduced survival [1,2]. The work on molecular profiling of invasive breast cancer has led to the identification of at least five distinct subtypes in which the most invasive and malignant type is entitled basal-like breast cancer [3]. This molecular subtype is predominantly oestrogen receptor alpha-negative, progesterone receptor-negative, human epidermal growth factor receptor 2-negative and EGFR-positive. The basal-like subtype is linked with poor clinical outcome and represents the most likely subgroup of breast tumours that could benefit from EGFR targeted therapy as they lack the other conventional receptor drug targets [3-5]. Similar to other receptor drug targets, however, clinical resistance to EGFR inhibitors or monoclonal antibodies is known to occur [6]. Developing alternative drug targets in the EGFR signalling pathway as means to treat EGFR-dependent invasive and metastatic breast cancer is therefore imperative.

Increased migration is a crucial component of increased invasion and metastasis of cancer cells. Key signalling molecules in the regulation of normal cell as well as cancer cell migration are the Rho GTPases, most notably Rho, Rac and Cdc42 [7]. Indeed, the acquisition of motile and invasive properties is a prerequisite to the development of a metastatic phenotype. These properties are dependent on the RhoGTPases, which are most widely recognised for their role in dynamic cytoskeletal remodelling [8,9]. RhoGTPases control diverse downstream actions through distinct effector proteins. Transfection of T47D breast cancer cells with constitutively active Cdc42 has been shown recently to drive migration via the Cdc42-specific effector TNK2 (formally known as Ack1), which binds to activated cdc42 but not to Rho or Rac, and subsequent activation of breast cancer antioestrogen resistance 1 (BCAR1) (formally known as p130Cas) [10,11]. (Some confusion has arisen in the literature regarding the nomenclature and identity of Ack1 – we herein refer to human Ack1 (NCBI Entrez GeneID 10188) as TNK2; it is not equivalent to Ack2, of which there is in fact no such human gene, but was originally the name of a bovine homologue of Ack1 [14].) TNK2 has also been suggested to function as an oncogene when overexpressed [12,13]. This hypothesis was supported by the finding that amplification of the TNK2 gene and mRNA, in primary tumours, correlates with poor prognosis [13].

Cdc42 has been linked previously with EGFR function. Cdc42 is proposed to function in a positive feedback loop with the EGFR whereby epidermal growth factor (EGF) stimulates activation of Cdc42 and its interaction with specific target proteins: Cdc42, in turn, inhibits EGFR degradation by preventing binding of c-Cbl to EGFR. This leads to aberrant accumulation of EGFR on the cell surface and subsequent malignant transformation [15].

Interestingly, TNK2 – a downstream effector of Cdc42 – can also be activated in response to EGF and interacts with EGFR via a previously characterised EGFR binding domain [16]. It has also been reported, however, that TNK2 regulates clathrin-mediated EGFR endocytosis and facilitates receptor degradation [17-19]. While Cdc42 maintains EGFR on the cell surface, therefore, TNK2 in contrast has paradoxically been reported to facilitate degradation, which is at odds with its potential role as an oncogene [15,20]. Importantly, no functional effects of the TNK2/EGFR interaction have been established in a cancer context to date – and, more importantly, it is not known how aberrant expression of EGFRs often found in cancer cells affects this protein–protein interaction.

In the present article we demonstrate the efficacy of targeting TNK2, a nonreceptor tyrosine kinase, by siRNA, and its effect on inhibiting EGFR cell surface expression and the migration and invasion of breast cancer cells. Significantly we found that silencing of BCAR1, a proposed downstream mediator of TNK2, inhibits breast cancer cell invasion via a mechanism distinct from the EGFR.

## Materials and methods

### Cell culture and transfection

MCF-7, MDA-MB-231 and MDA-MB-468 breast cancer cells (American Type Culture Collection, Manassas, VA, USA) were cultured in DMEM supplemented with 10% FBS, 500 U/ml penicillin 500 μg/ml, and 2 mM L-glutamine. Transient transfection of siRNA was carried out using Lipofectamine 2000 (Invitrogen, Carlsbad, CA, USA). Subconfluent cells were washed twice in PBS and once in Optimem (Invitrogen, Carlsbad, CA, USA) medium, and were incubated with a complex of Lipofectamine 2000 and siRNA in Optimem (at a final concentration of 100 nM siRNA) for a period of 3 hours. Cells were then washed twice in PBS and the normal antibiotic and FBS-containing DMEM medium was replaced.

Subsequent experiments were carried out a minimum of 48 hours following transfection to ensure efficient silencing of the targeted protein. For all subsequent assays performed, downregulation of the protein of interest by siRNA was ensured by western blot analysis. For plasmid transfection, the procedure was the same except that subsequent experiments were carried out from 24 hours post-transfection. The wildtype, kinase-deficient and constitutively active Wt-TNK2, Ca-TNK2 and Kd-TNK2 constructs were kindly provided by Takaya Satoh (Kobe, Japan) [21].

### Reagents

Antibodies were obtained from the following: mouse monoclonal TNK2 (1/300) (Santa Cruz Biotechnology, Santa Cruz, CA, USA); fluorescein isothiocyanate-conjugated rat monoclonal EGFR for fluorescence-activated cell sorting (FACS) analysis (1/100) (Abcam, Cambridge, UK); mouse monoclonal EGFR (1/1,000), mouse monoclonal p-EGFR (1/1,000) and mouse monoclonal BCAR1 (1/2,000) (BD Biosciences, Bedford, MA, USA); mouse monoclonal β-actin (1/25,000) (Sigma-Aldrich, Saint Louis, MO, USA); and Alexa-Fluor 488 Phalloidin (1/500) (Invitrogen, Carlsbad, CA, USA). Recombinant human EGF and the caspase substrate Ac-DEVD-amc were purchased from Upstate (Lake Placid, NY, USA).

Predesigned siRNAs targeting human TNK2 (#103419, #124657) and BCAR1 (#21608, #21699) and nontargeting negative control siRNA (#4611) were purchased from Ambion (Cambridgeshire, UK). The Biocoat Matrigel Invasion assays were purchased from BD Biosciences. PD153035 was purchased from Calbiochem (La Jolla, CA, USA). Alamar Blue reagent for proliferation assay was purchased from Serotec (Oxford, UK). 4',6-Diamidino-2-phenylindole was purchased from Sigma. Hoechst 34580 was purchased from Invitrogen.

### Immunoblotting and immunoprecipitation

For western blotting, cultured cells were lysed directly in Laemmli buffer with dithiothreitol and were boiled. For immunoprecipitation, cells were scraped into PBS and the cell pellet was then lysed with buffer containing 100 mM Tris–HCl (pH 7.5), 1% Triton X-100, 5 mM ethylenediamine tetraacetic acid, 5 mM EGTA, 50 mM NaCl, 4 mM Na_3_VO_4_, 20 μg aprotinin/ml, 1 μg leupeptin/ml, 2.5 mM benzamidine, and 2 mM Pefabloc (Roche, Basel, Switzerland). The lysates were precleared with protein-A Sepharose for 1 hour at 4°C, and immunoprecipitations were performed with beads preconjugated with the immunoprecipitating antibody with constant agitation for 2 hours at 4°C. The beads were washed five times in ice-cold wash buffer (50 mM HEPES (pH 7.4), 1% Triton X-100, 0.1% SDS, 150 mM NaCl, 2 mM Na_3_VO_4_) and were boiled immediately in Laemmli buffer with dithiothreitol.

Proteins were separated by SDS-PAGE under reducing conditions and were then transferred to polyvinyldifluoride membranes by electroblotting. The membranes were blocked with 4% powdered milk in PBS 0.1% Tween 20 (PBS-Tween) at room temperature (RT) for 30 minutes and then probed with primary antibodies diluted in 2% powdered milk in PBS-Tween overnight at 4°C or 2 hours at RT. The membranes were then washed three times with PBS-Tween and probed with horseradish peroxidase-conjugated secondary antibodies at 1:10,000 dilutions in 4% powdered milk in PBS-Tween for 1 hour at RT. Following washing three times with PBS-Tween, the membranes were developed with the enhanced chemiluminescence western blotting detection system (Pierce Biotech, Rockford, IL, USA).

### Immunocytochemistry

Following siRNA transfection (at least 48 hours), cells were transferred to chamber slides (BD Biosciences) and were allowed to adhere overnight. Cells were then washed in PBS and fixed in 4% paraformaldehyde for 10 minutes at RT. The cells were then again washed in PBS and permeabilised in 0.5% Triton-X in PBS for 5 minutes at RT. Cells were then incubated in a blocking solution of 3% BSA/PBS-Tween until staining was performed. Actin filaments were stained with Alexa-Fluor 488-conjugated Phalloidin (1/500) in blocking solution at 4°C overnight or for 2 hours at RT. Following washing five times with PBS-Tween, cell nuclei were stained with 10 μg/ml 4',6-diamidino-2-phenylindole in blocking solution for 10 minutes at RT. Cells were washed for a final time with PBS before coverslips were mounted with a fluorescence mounting medium and the slides were photographed.

### Apoptosis assays

#### Caspase-3 assay

Cells for analysis per timepoint were divided into 60 mm culture dishes. At the indicated timepoints, cells were harvested with 200 μl caspase lysis buffer (10 mM Tris–HCl, pH 7.4, 10 mM NaH_2_PO_4_/Na_2_HPO_4_, pH 7.4, 130 mM NaCl, 0.1% Triton-X, and 10 mM NaPP_i_). Floating cells were collected and pooled with the lysate. The lysate was divided to perform the assay in triplicate in a 96-well plate. The lysate protein concentration was measured to ensure equal amounts of protein were used. To each sample well, 50 μl lysate, 150 μl reaction buffer (20 mM HEPES, pH 7.5, 10% glycerol, and 2 mM dithiothreitol), and 3 μl caspase substrate Ac-DEVD-amc in dimethylsulfoxide (Upstate) was added. The reaction mixture was incubated at 37°C for 1 hour, and thereafter fluorescence was measured with a Fluostar plate reader (BMG Lab Technologies, Offenburg, Germany) using excitation and emission wavelengths of 390 nm and 460 nm, respectively.

#### Hoechst staining

Cells were seeded onto glass coverslips and, at the indicated timepoints, were washed once with PBS, fixed for 15 minutes in 4% paraformaldehyde, washed again with PBS, and then incubated with Hoechst 34580 at a final concentration of 5 μg/ml at RT for 10 minutes. Following the staining procedure, cells were washed with PBS before coverslips were mounted with a fluorescence mounting medium (Dako, Glostrup, Denmark). Nuclear morphology was examined and 200 cells were counted per treatment.

### Proliferation assay

A sample of 1,000 MDA-MB-231 cells or 2,000 MCF-7 cells in 196 μl DMEM (containing 10% serum or serum free) with or without EGF (100 ng/ml) were seeded in each well of a 96-well plate. Alamarblue (4 μl) was then added directly to the wells. These plates were incubated for 2 hours at 37°C before making the initial measurement (timepoint 0). Fluorescence was measured using excitation and emission wavelengths of 540 nm and 590 nm, respectively.

### Epidermal growth factor receptor internalisation analysis

Cells were serum-starved overnight (16 hours), followed by EGF (100 ng/ml) stimulation for the time periods specified at 37°C. Following this treatment, cells were detached using Versene (Invitrogen), and washed in FACS buffer (PBS containing 0.5% BSA and 2 mM ethylenediamine tetraacetic acid) while incubated on ice. Fluorescein isothiocyanate-conjugated EGFR antibody was added to the cells resuspended in 100 μl FACS buffer for a period of 1 hour in the dark at 4°C. One sample to be used as a negative control for background signal was not incubated with antibody. The cells were then washed twice in 2 ml FACS buffer and resuspended in a volume of 300 to 600 μl for analysis. The sample remained on ice until the end of the procedure. Samples were run on the BD Facscalibur system (BD Biosciences, Bedford, MA, USA) and the data analysed using CellQuest software (BD Biosciences). The percentage of cell surface receptors was calculated by setting the value for the negative control siRNA (N) at 100% cell surface receptor at timepoint 0; the other values were extrapolated from this value.

### Migration assay

Cells were grown to confluence, scratched with a pipette tip, and washed twice in PBS to remove floating cells. When the EGFR inhibitor PD153035 (1 μM) was used, cells were treated for 60 minutes prior to the addition of EGF (100 ng/ml). As the wound healed over a period of up to 48 hours depending on the cell type, the cells were photographed at intervals using an inverted microscope; the sizes of the wounds were subsequently analysed with the Image J program, 1.37v (National Institutes of Health, Bethesda, MD, USA).

### Invasion assay

Cells were counted 48 hours post transfection and equal numbers were added to invasion chambers essentially as described in the manufacturer's protocol (Biocoat Matrigel Invasion; BD Biosciences). Invasion typically proceeded over 48 hours, and the cells were stained and counted thereafter as described. Lysates of the cells used for the invasion assays at the beginning and end of the experiment were taken for western blot analysis to ascertain the efficiency of the siRNA transfection in each case.

### Statistical analysis

Statistical analyses were performed using Microsoft Excel. Statistical significance was determined using a two-tailed Student's *t *test. Replicates in the assays used are biological replicates representing repetition of the experiments following a minimum of three separate transfections or treatments.

## Results

### Downregulation of TNK2 by siRNA in human breast cancer cells results in unique cytoskeletal and morphological changes

**Figure 1:**
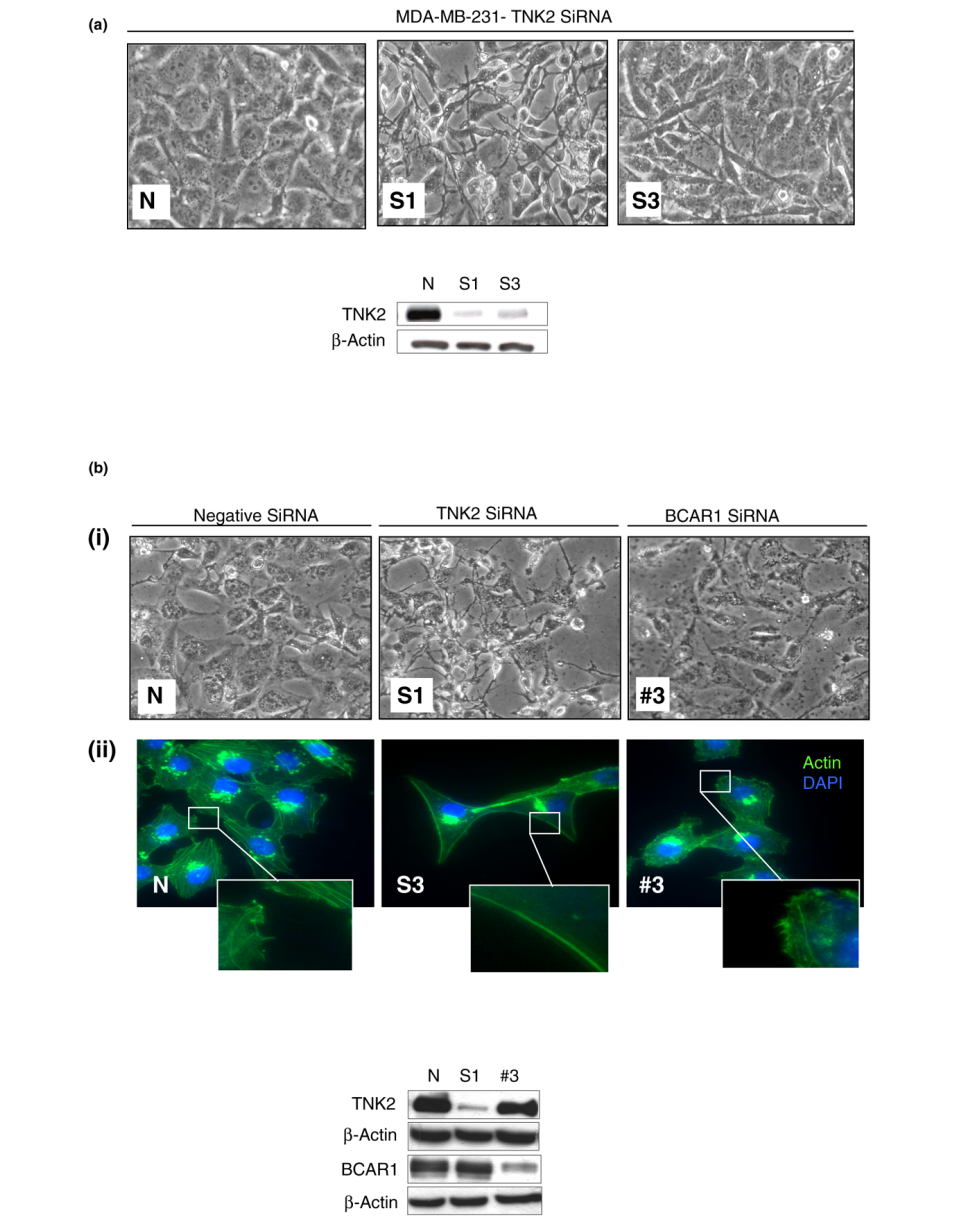
**Downregulation of TNK2 by siRNA induces morphological and cytoskeletal changes in human breast cancer cells**. **(a) **MDA-MB-231 cells treated with TNK2-targeting SiRNAs (S1, S3) were observed to undergo morphological changes after transfection relative to the nontargeting SiRNA control (N). These changes consisted of an increased elongated appearance and a reduction in the number of protrusions at the membrane edges. A representative western blot illustrating the downregulation of TNK2 achieved by siRNA (S1, S3) treatment relative to β-actin is shown. **(b) **The morphology changes observed in TNK2 SiRNA-treated cells were not observed in MDA-MB-231 cells treated with SiRNA directed against breast cancer antioestrogen resistance 1 (BCAR1). (i) Cells transfected with nontargeting (N), TNK2-targeting (S1) and BCAR1-targeting (#3) siRNA. (ii) The morphological changes induced can also be seen as an alteration in the actin fibre network. Staining for F-actin and costaining for the nuclear compartment with 4',6-diamidino-2-phenylindole is shown for cells treated with nontargeting (N), TNK2-targeting (S3) and BCAR1-targeting siRNA (#3). A representative western blot illustrating the downregulation of TNK2 (S1) and BCAR1 (#3) achieved by SiRNA treatment relative to β-actin is shown.

Initially, upon treatment of MDA-MB-231 breast cancer cells with siRNA directed against TNK2, we observed morphological changes by 48 hours post transfection – indicative of cytoskeletal remodelling – that were characterised by an increasingly elongated stellate appearance with a distinct absence of protrusions at the membrane edges (Figure 1a). A representative western blot is shown illustrating siRNA downregulation of TNK2 (S1, S3) relative to the control (N). Surprisingly, however, we noted that these changes were not recapitulated upon treatment with siRNA targeting the proposed TNK2 downstream effector, BCAR1. This observation indicated that the effects induced by TNK2 were independent of BCAR1 (compare TNK2 siRNA (S1) and BCAR1 siRNA (#3) against the nontargeting control (N) in Figure 1b,i).

Staining for F-actin revealed that TNK2-silenced cells exhibited smooth, straight actin bundles at their membrane edges (S3 and inset in Figure 1b,ii) in comparison with the protruding, uneven edges of normal cells or BCAR1-silenced cells (N and #3 in Figure 1B,ii). A representative western blot is shown illustrating siRNA downregulation of TNK2 (S1) and BCAR1 (#3) relative to the control (N). Given the previous reported role of TNK2 in EGFR dynamics [17,19] and the potential impact of EGFR activation on migration and cytoskeleton remodelling [22,23], we hypothesised that the EGFR might be involved in the mechanism of action of TNK2 in breast cancer cells and hence be related to the observed morphological phenotype.

### TNK2 binds EGFR in a TNK2-kinase-independent manner, and the association is enhanced by EGFR activation

**Figure 2:**
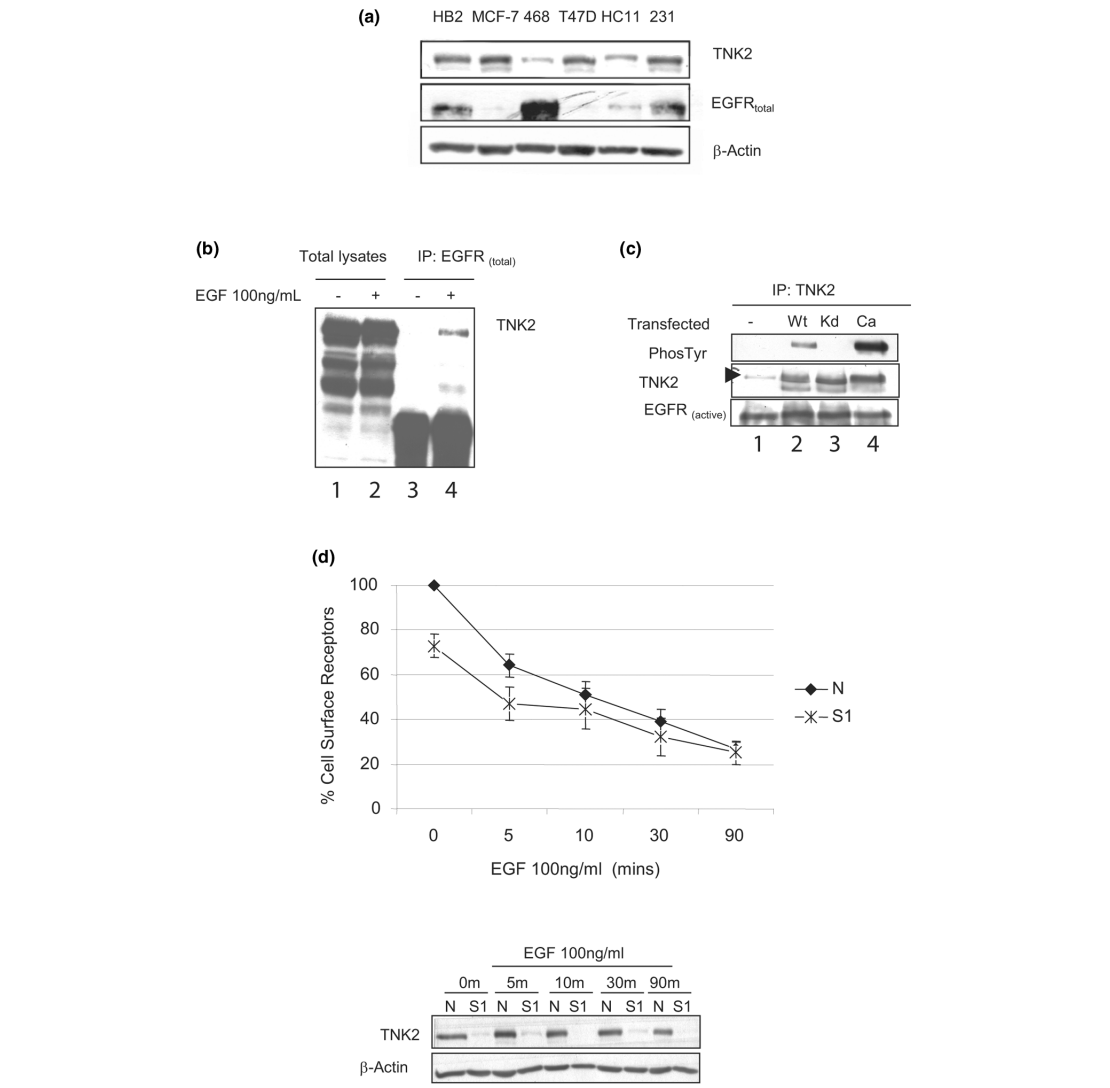
**Epidermal growth factor treatment of breast cancer cells promotes the interaction of TNK2 with EGFR**. **(a) **Epidermal growth factor receptor (EGFR) and TNK2 have variable protein expression levels in normal and breast cancer cell lines. The levels of TNK2 are shown relative to β-actin protein levels in the following cell lines: HB2, normal human breast epithelial cells; MCF-7, MDA-MB-468, and T47D human breast cancer cells; HC11 murine mammary epithelial cells; and human breast cancer cell line, MDA-MB-231. **(b) **TNK2 is recruited to the EGFR following activation of the receptor in MDA-MB-231 cells, as indicated by coimmunoprecipitation in the presence and absence of epidermal growth factor (EGF) stimulation at 100 ng/ml. **(c) **The association of EGFR and TNK2 in MDA-MB-231 cells is not dependent on the phosphorylation status of TNK2, as indicated by the ability of wildtype (Wt), mutant kinase-deficient (Kd) and constitutively active (Ca) TNK2 to immunoprecipitate activated EGFR. **(d) **The percentage cell surface expression of EGFR is shown in serum-starved MDA-MB-231 cells treated with EGF (100 ng/ml) for the indicated times. Reduced numbers of basal cell surface EGFRs are seen in cells treated with targeting SiRNA (S1) relative to the nontargeting SiRNA control (N), as measured by a fluorescein isothiocyanate-conjugated anti-EGFR antibody by fluorescence-activated cell sorting analysis. On average, a 27% reduction of cell surface receptors can be seen at timepoint 0 (*P *= 0.0135). A representative western blot illustrating the downregulation of TNK2 achieved by SiRNA treatment relative to β-actin is shown.

We examined a range of breast cancer cell lines with varying levels of EGFR and TNK2. The total protein levels of both TNK2 and EGFR are shown in Figure 2a; MCF-7 cells have moderately low EGFR levels relative to MDA-MB-231 cells, which overexpress EGFR but lack EGFR gene amplification in contrast to the MDA-MB-468 cell line, which has both genomic amplification and overexpression of EGFR [24]. We could demonstrate in serum-starved cells that endogenous TNK2 binding to endogenous EGFR was enhanced by EGFR activation (Figure 2b), although the ability of TNK2 to bind EGFR in normal serum, without EGF stimulation, was also evident (Figure 2c, lane 1). Furthermore, we found that cells transfected with wildtype, kinase-deficient or constitutively active TNK2 have equal ability to immunoprecipitate active EGFR (Figure 2c, lanes 2 to 4). This observation demonstrates the novel finding that, although TNK2/EGFR interaction may be influenced by EGFR activation, it appears to be independent of TNK2 kinase activity.

Downregulation of TNK2 by siRNA reduces the number of cell surface EGFRs. To investigate the functional consequences of the observed TNK2/EGFR interaction, we wanted to examine how EGFR dynamics might be affected in cancer cells in which TNK2 had been silenced by siRNA treatment. In particular, we wanted to investigate the effect on cell surface EGFRs, as this population of receptors is responsible for initiation of signalling in response to extracellular ligands. Additionally, this cell surface population has not been examined in previous reports, which investigated only the total intracellular levels of EGFR. Accordingly, MDA-MB-231 cells were serum-starved overnight, incubated with 100 ng/ml EGF for up to 90 minutes and analysed by flow cytometry (FACS) to determine the relative number of cell surface EGFRs using a fluorescein isothiocyanate-conjugated antibody.

Although there was little difference in the ability of the TNK2-silenced cells relative to nontargeting siRNA control cells (N) to internalise EGFR in response to ligand (Figure 2d; both values fall when exposed to EGF), we found that there was in fact a significantly reduced number of basal cell surface EGFRs (timepoint 0) in TNK2-silenced cells (S1) relative to nontargeting siRNA control cells (N) (Figure 2d). One caveat is that if we take the relative rates by defining the percentage of receptors lost over the 90 minutes, there appears to be a slightly slower rate of internalisation in the TNK2 siRNA-treated cells. This finding argues that increased internalisation may not be the mechanism that leads to decreased cell surface EGFR. Of course, the finding may also simply reflect the fact that, in these cells, a reduced cell surface population results in a reduced internalisation rate. The experiment was repeated four times and represents four separate transfections. On average, a 27% reduction of cell surface receptors can be seen at timepoint 0 (*P *= 0.0135) prior to any ligand stimulation. A representative western blot is shown illustrating siRNA downregulation of TNK2 (S1) relative to the control (N) over the course of the experiment.

### EGFR activation enhances the migration of breast cancer cells with high and low EGFR expression

**Figure 3:**
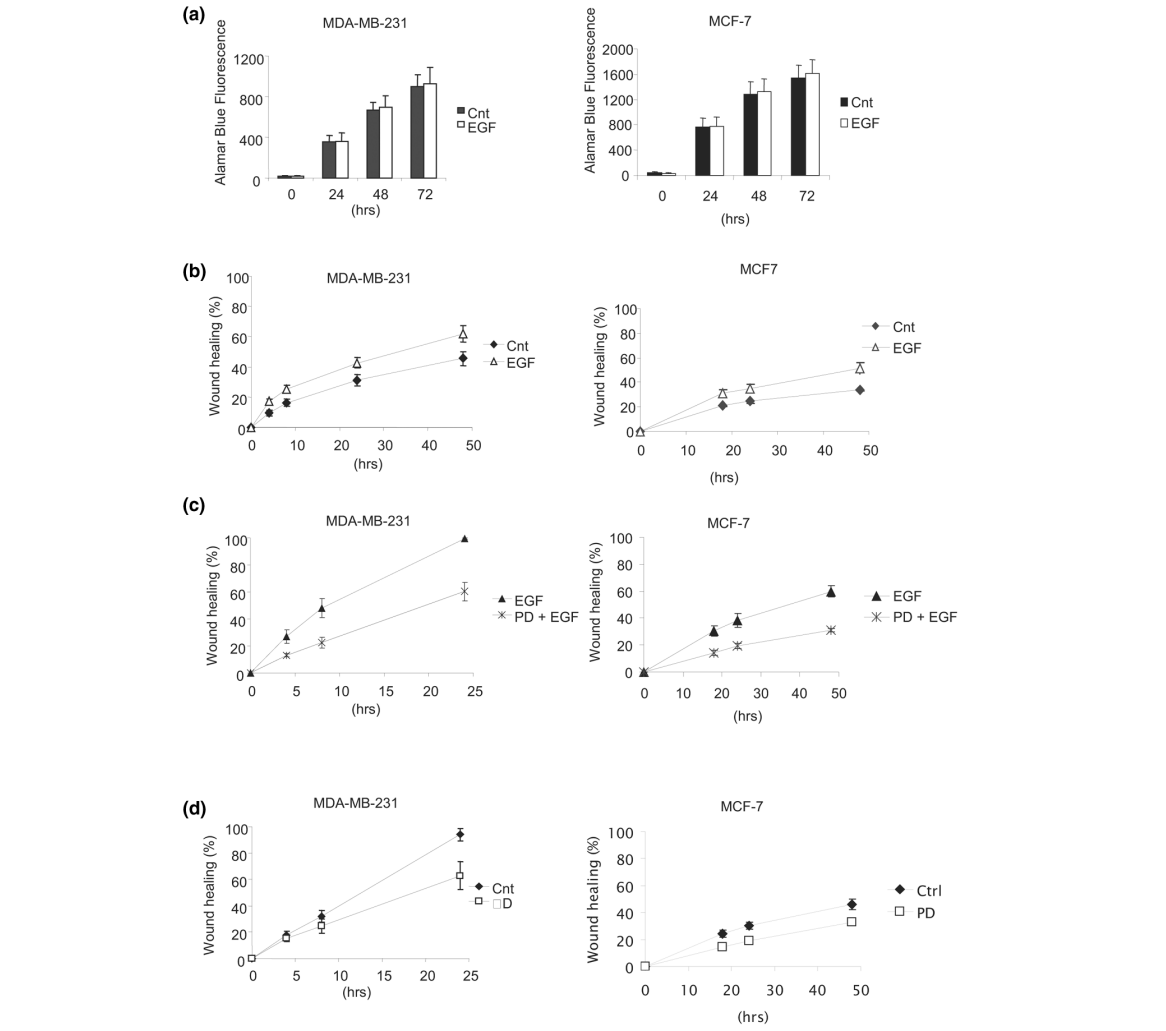
**Epidermal growth factor treatment promotes the migration but not the proliferation of breast cancer cells**. **(a) **Proliferation as measured by an Alamar Blue proliferation assay was not significantly affected in serum-starved cells of either cell type over a period of 72 hours. **(b) **Epidermal growth factor (EGF) treatment (100 ng/ml) enhanced MDA-MB-231 cell and MCF-7 cell migration as measured by a scratch-wound migration assay. Healing due to cell migration was measured over a period of 48 hours following wounding. The difference in migratory ability induced by EGF was significant in both cell types (*P *= 0.0483 and *P *= 0.013 at 48 hours for MDA-MB-231 cells and MCF-7 cells, respectively). **(c) **Treatment of both EGF-treated MDA-MB-231 and EGF-treated MCF-7 cells with the epidermal growth factor receptor kinase inhibitor PD153035 (PD) significantly inhibited wound healing in serum-containing medium (*P *= 0.0004 and *P *= 0.0006 at the end points, respectively). **(D) **Treatment of both MDA-MB-231 cells and MCF-7 cells with PD significantly inhibited wound healing in serum-containing medium (*P *= 0.028 at 24 hours and *P *= 0.0264 at 48 hours, respectively).

EGFR activation at the plasma membrane has been proposed to control or to contribute towards a multitude of cell processes in normal and cancerous cells, including proliferation and migration [25]. Because of the effect of TNK2 siRNA on the cell surface EGFR population, we wanted to determine the effect of EGFR activation on breast cancer cell behaviour. Interestingly, we could demonstrate that addition of EGF to serum-starved cells (or nonstarved cells, data not shown) had no significant effect on the rate of proliferation over 72 hours as shown by an Alamar Blue proliferation assay (Figure 3a). In contrast, we show that activation of EGFR in breast cancer cells significantly alters the motile properties of these cells. This was true of cells with high (MDA-MB-231) or low (MCF-7) relative levels of EGFR. MDA-MB-231 and MCF-7 cells were allowed to grow to a confluent monolayer, after which a scratch wound was made using a pipette tip. Migration was measured as the percentage area refilled over the time periods, as indicated in Figure 3. The percentage of increased migration with the addition of EGF was found to be significant in both cell types (MDA-MB-231 cells, *P *= 0.0483 and MCF-7 cells, *P *= 0.013 at 48 hours in serum-free medium; Figure 3a).

We also confirmed that EGFR activity was responsible for the effects on migration by use of an EGFR reversible tyrosine kinase inhibitor, PD153035 [26]. In accordance with our hypothesis, EGF-stimulated migration was significantly reduced by PD153035 in both MDA-MB-231 and MCF-7 breast cancer cells (*P *= 0.0004 and *P *= 0.0006 at the end points, respectively; Figure 3c). Inhibition of basal migration in the presence of PD153035 was also demonstrated in the absence of additional exogenous EGF. The activation of the EGFR even by serum-supplemented medium alone therefore significantly altered the motility of MDA-MB-231 and MCF-7 breast cancer cells (*P *= 0.028 and *P *= 0.0264 at the end points, respectively; Figure 3d).

### Reduced cell surface EGFR expression induced by TNK2 siRNA correlates with reduced migration, but not proliferation or apoptosis

**Figure 4:**
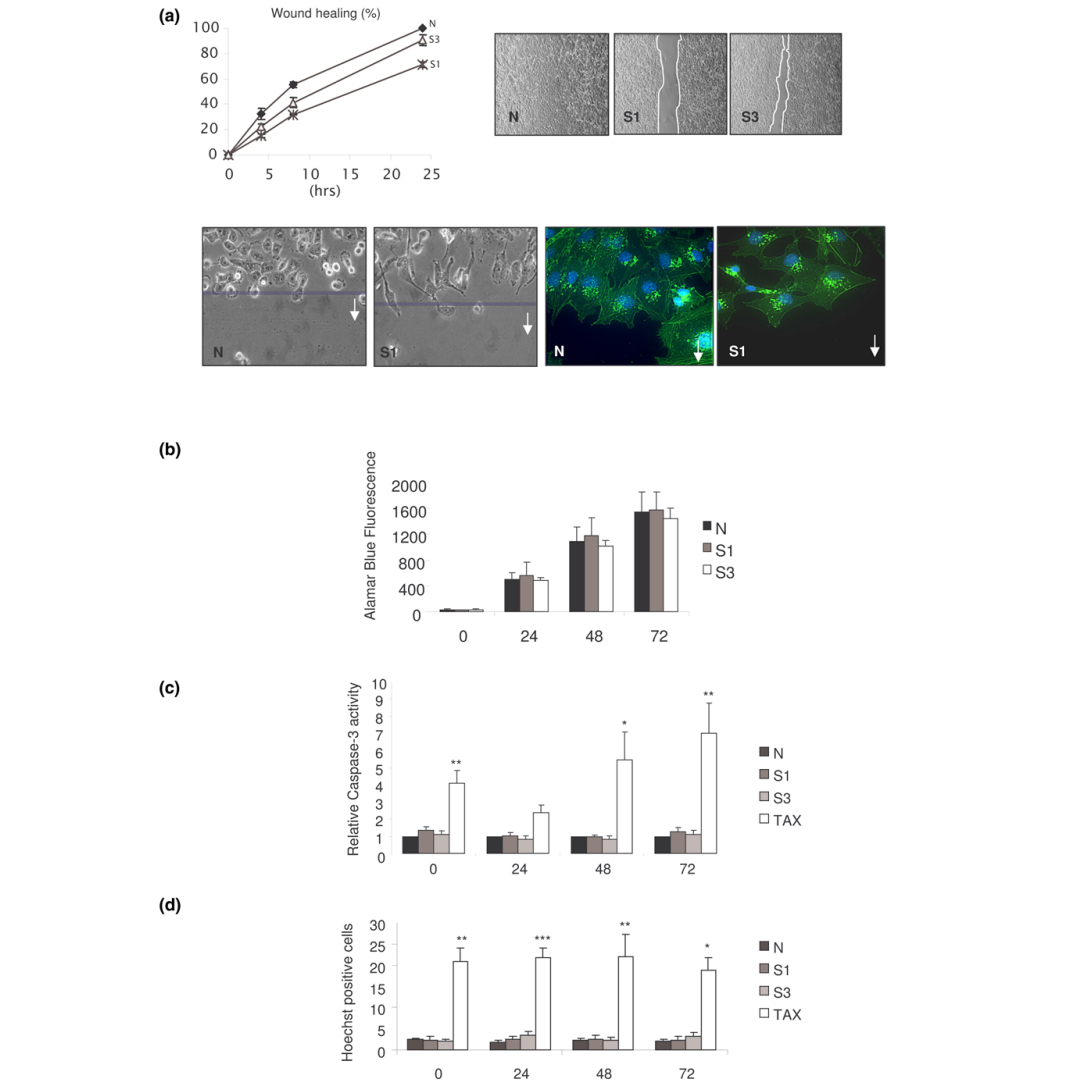
**Downregulation of TNK2 by siRNA inhibits human breast cancer cell migration**. **(a) **MDA-MB-231 cells treated with TNK2 targeting SiRNA (S1, S3) had less ability to migrate relative to the nontargeting SiRNA control (N) as measured by a scratch-would healing assay. There was a significant difference between nontargeting SiRNA control (N) and TNK2-targeting SiRNA (S1) at 4, 8 and 24 hours (*P *= 0.0195, *P *= 0.0004 and *P *= 0.0002, respectively). There was a significant difference between nontargeting SiRNA control siRNA (N) and TNK2-targeting SiRNA (S3) at 8 hours (*P *= 0.032). Lower panels: representative pictures of the scratch-would assays 48 hours following wounding. **(b) **Proliferation as measured by an Alamar Blue assay was not significantly affected in cells treated with the TNK2-targeting SiRNA (S1, S3) relative to the nontargeting control siRNA (N) over a period of 72 hours. **(c) **Caspase-dependent apoptosis as measured by a caspase-3 cleavage assay showed no significant difference between cells treated with the TNK2-targeting SiRNA (S1, S3) relative to the nontargeting control siRNA (N) over a period of 72 hours. Taxol is used as a positive control (TAX) and showed significant differences in all cases. **(d) **There was no significant difference seen for caspase-independent apoptosis as measured by Hoechst staining between cells treated with the TNK2-targeting SiRNA (S1, S3) relative to the nontargeting control siRNA (N) over a period of 72 hours. Taxol is used as a positive control (TAX) and showed significant differences in all cases. *P ≤ 0.05, **P ≤ 0.01, ***P ≤ 0.001.

Given that the suppression of TNK2 resulted in reduced cell surface expression of EGFR (Figure 2d), and that activation of cell surface EGFR is responsible for migration (Figure 3), we hypothesised that reduced cell surface expression of EGFR, induced by TNK2 siRNA, should also result in reduced migration but should not affect apoptosis or proliferation. We consequently investigated the migratory capacity of MDA-MB-231 and MCF-7 cells transfected with targeting siRNA (S1, S3) and nontargeting siRNA (N) using the scratch-wound migration assay. Migration was slower in cells transfected with targeting siRNA (S1, S3) than control nontargeting siRNA (N), demonstrating that silencing of TNK2 inhibits human breast cancer cell migration (Figure 4a for MDA-MB-231 cells; Additional file 1A for MCF-7 cells). Furthermore, as expected, there was no significant difference in an Alamar Blue proliferation assay between the targeting (S1, S3) siRNA-treated cells and the nontargeting (N) siRNA-treated cells (Figure 4b).

Additionally, caspase-3 activity and Hoechst staining assays performed indicated no significant differences between the targeting and nontargeting control in the amount of cells undergoing apoptosis (Figure 4c,d). These results are consistent with our above observation that the function of EGFR activation is limited to effects on migration, and verify that increased apoptosis or decreased proliferation is not responsible for the reduction in migration seen.

### BCAR1 siRNA results in reduced invasion without causing a reduction in cell surface EGFR expression

**Figure 5:**
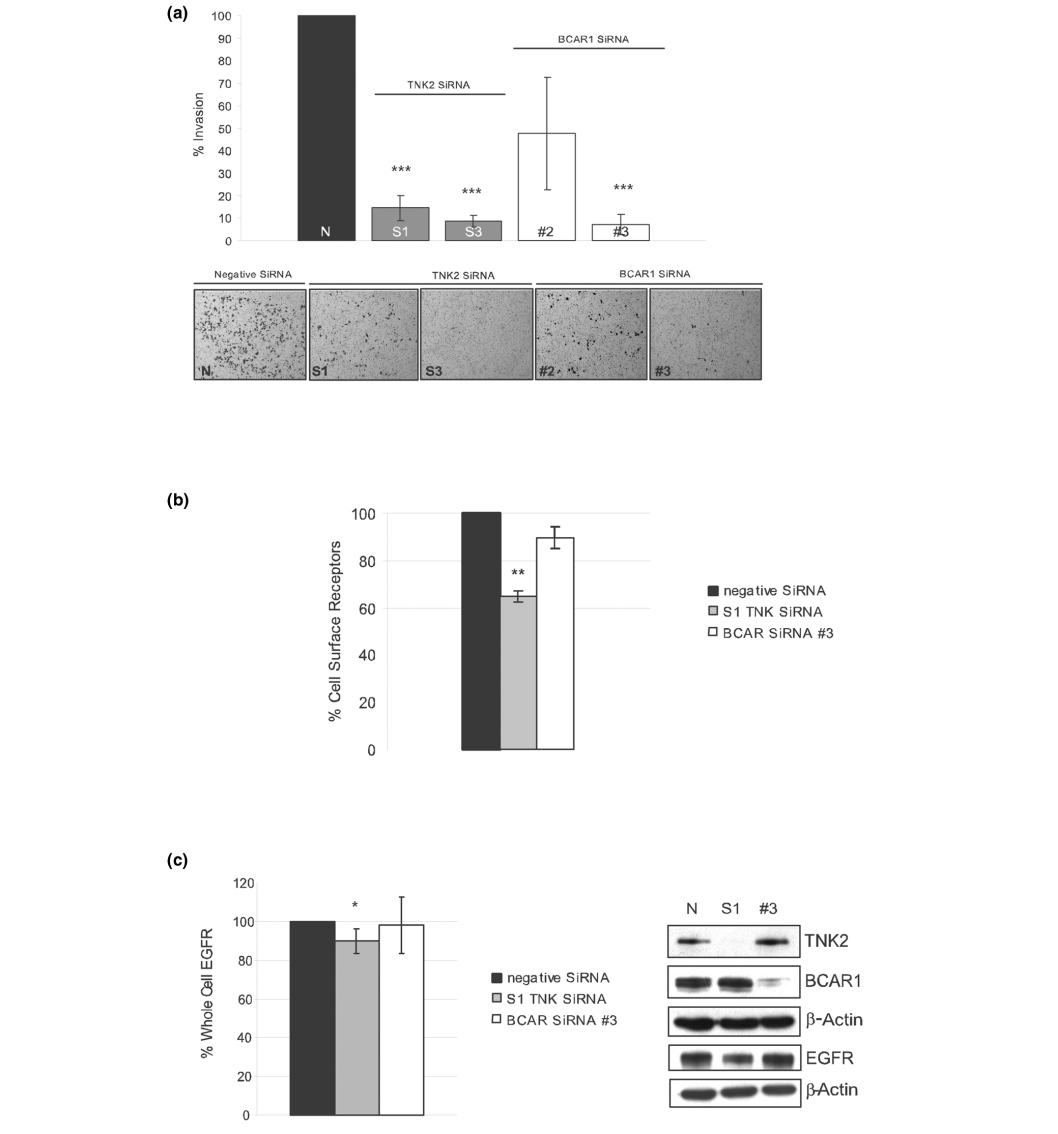
**TNK2 controls and BCAR1 control breast cancer cell invasion via distinct mechanisms**. **(a) **Invasion assays were carried out using MDA-MB-231 cells transfected with targeting siRNA (S1, S3) and nontargeting SiRNA (N). At 48 hours post transfection, cells were detached, counted, added to modified Boyden chambers coated with Matrigel, and allowed to invade over a period of 48 hours. Following this, cells that had invaded were fixed *in situ*, stained and counted. The assay was repeated a total of six times, revealing an average inhibition of invasion of 85.5%, *P *< 0.0001 (S1) and 91.2%, *P *< 0.0001 (S3) relative to the nontargeting SiRNA control (N). A similar effect on the invasive capacity of MDA-MB-231 cells was seen in cells treated with SiRNA targeting breast cancer antioestrogen resistance 1 (BCAR1) silencing, where the average inhibition of invasion was 52.5%, *P *= 0.0628 (#2) and 93%, *P *< 0.0001 (#3). A representative image is shown illustrating the cells that had invaded the Matrigel over the course of the invasion assay. **(b) **Effect of targeting TNK2 SiRNA (S1) versus BCAR1 SiRNA (#3) and nontargeting SiRNA (N) on the levels of cell surface epidermal growth factor receptors (EGFRs) as measured by fluorescence-activated cell sorting analysis. On average, a 35% reduction of cell surface receptors can be seen at timepoint 0 (*P *= 0.0037) in cells treated with SiRNA targeting TNK2 (S1); however, the reduction seen with BCAR1 was not significant (*P *= 0.156).**(c) **Effect of TNK2 siRNA treatment on the total cellular EGFR expression as measured by western blot analysis. Approximately a 10% reduction in total EGFR is observed with TNK2 siRNA treatment. There is no statistically significant reduction seen with BCAR1 siRNA treatment. A representative western blot illustrating the downregulation of TNK2 (S1) and BCAR1 (#3) achieved by SiRNA treatment and their respective EGFR levels relative to β-actin is shown. *P ≤ 0.05, **P ≤ 0.01, ***P ≤ 0.001.

Inducing an alteration in the motile properties of cells does not necessarily reflect an alteration in the invasive capacity of such cells or indeed reflect a reduced potential to metastasis *in vivo*. The ability to demonstrate a reduced propensity to invade surrounding tissue is therefore paramount for *in vivo *relevance. Accordingly, we investigated the invasive capacity or metastatic tendency of these cells using a modified Boyden chamber coated with Matrigel. At 48 hours following siRNA transfection, cells were detached, counted, added to the modified Boyden chambers and allowed to invade the Matrigel-coated filter for a further 48 hours. The assay was repeated a total of six times (representing six separate transfections) for MDA-MB-231 cells, revealing an average inhibition of invasion of 85.5% (S1) and 91.2% (S3) relative to the nontargeting siRNA control (N) (Figure 5a). The assay was also performed on MCF-7 cells (n = 6), revealing an average inhibition of invasion of 33.5% (S1) and 32.7% (S3) relative to the nontargeting siRNA control (N) (see Additional file 1B).

Interestingly, we noted that MDA-MB-231 cells treated with BCAR1 siRNA also showed a reduced invasive capacity consistent with the assumption that BCAR1 can regulate cell migration downstream of TNK2 as previously proposed [10]. An invasion chamber assay experiment revealed an average inhibition of 52.5% (#2) and 93% (#3) in MDA-MB-231 cells (Figure 5a). Crucially, however, we found that downregulation of BCAR1 by siRNA, unlike TNK2 siRNA, did not have a significant effect on basal cell surface EGFR expression (Figure 5b).

We also found that the total cellular amount of EGFR was to a small (~10%), but statistically significant, extent reduced in response to TNK2 siRNA treatment as examined by western blot analysis at the same timepoint as when reduced cell surface EGFR expression was observed. The graph (Figure 5c) represents densitometry analysis of six separate transfections. There was no difference in the total cellular amount of EGFR in BCAR1 siRNA-treated cells. A representative western blot is shown, illustrating relative total protein levels in the samples investigated (Figure 5c).

## Discussion

Initially, we observed that targeting of TNK2 by siRNA in human breast cancer cells resulted in distinct cytoskeletal and morphological changes, potentially indicative of changes in the motile properties of these cells. Such changes were not seen upon siRNA targeting of its proposed downstream effector BCAR1. This finding led us to hypothesise that the observed cytoskeletal effects induced by TNK2 must be independent of BCAR1. We subsequently observed that TNK2 associates with activated EGFR in breast cancer cells in a TNK2-kinase-independent manner, and furthermore that it functions to maintain EGFRs on the cell surface. We contend that this observation implies TNK2 may ordinarily function downstream of Cdc42 in the reported positive feedback loop whereby activated Cdc42 maintains cell surface EGFR expression [15]. This effect of Cdc42 on EGFR stability has been previously shown to contribute to enhanced cell migration by activated Cdc42 [20]. Our data now indicate that the same is true for TNK2, since the significant reduction of cell surface EGFRs we observed by TNK2 silencing was accompanied by a parallel decrease in the migratory capacity of the breast cancer cells. We also show that TNK2 siRNA has the same effect on invasion. In contrast, however, there is no affect of TNK2 siRNA on proliferation or apoptosis, which is in agreement with our findings that the main functional effect of EGFR activation in these breast cancer cells is stimulation of motility.

Previous studies claiming that TNK2 functions to promote degradation of EGFR appear to be at odds with the functional role of TNK2 *in vitro *and *in vivo *and with the results we now present. One important caveat, however, is that these previous studies examined total receptor expression in cleared cell lysates, which does not account for changes in the detergent insoluble cytoskeletal bound EGFR fraction [17,19]. The cytoskeleton or actin-bound EGFR fraction is reported to comprise the type I, high-affinity EGFRs that are primarily responsible for induction of cellular responses to ligand stimulation at the cell surface [27-29]. As such, it is imperative that any study analysing changes in EGFR levels include the cytoskeletal-bound EGFR fraction. Our results show that the total EGFR content, including the detergent-insoluble cytoskeletal fraction, is in fact slightly reduced with TNK2 siRNA treatment. The reduction of EGFR in the whole cell amounts to ~10%, whereas there is between 27% and 35% of cell surface receptors lost from the cell surface population. The percentage lost from the surface is higher than the average percentage lost from the whole cell, indicating that there is actually a selective reduction in cell surface EGFRs induced by TNK2 siRNA treatment and that the reduced cell surface receptor content is not solely as a result of increased EGFR degradation.

In the present study we have established that, even if constitutively active Cdc42 has not been introduced into the cells, TNK2 silencing alone is sufficient to both inhibit migration and to reduce the amount of EGFR on the cell surface. We also show here that BCAR1 siRNA silencing can function to inhibit invasion of breast cancer cells, even when the cells were not transfected with constitutively activated Cdc42 as was previously demonstrated [10]. Importantly, however, we show that BCAR1 silencing does not effect EGFR basal cell surface expression, demonstrating a distinct and independent effect of TNK2. This confirms our hypothesis that TNK2 can operate separately from BCAR1 to facilitate migration and invasion of breast cancer cells. Finally, these independent mechanisms and disparate effects can also explain the discrepancy in the morphological changes we observed following TNK2 and BCAR1 siRNA treatments. As EGFR activation can directly induce morphological changes via cytoskeleton remodelling, this supports our assertion that the morphological changes we see with TNK2 but not with BCAR1 siRNA treatment can be related to the ability to of TNK2 to affect the EGFR.

A prerequisite for classifying a molecule as a target for pharmacological intervention is demonstrating not only that it possesses oncogenic properties, but that abolition of its activity, by selective targeting, actually causes a positive anticancer effect that could be potentially useful in the treatment of disease. We have now established the potential of TNK2 in this regard by a siRNA-silencing approach. It is also of interest to note the effectiveness of TNK2 silencing in suppressing migration of not only cells that highly overexpress EGFR, such as MDA-MB-231 cells, but also those that do not overexpress EGFR but harbour functional EGFRs, such as the MCF-7 cells. While both MCF-7 and MDA-MB-231 cells expressed appreciable amounts of TNK2, MCF-7 cells are oestrogen-responsive breast cancer cells and have low levels of EGFR while MDA-MB-231 breast cancer cells do not express oestrogen receptor alpha, progesterone receptor or human epidermal growth factor receptor 2 but have very high levels of EGFR. Both cell lines, as we have shown, respond to EGFR activation by increased migration. MDA-MB-231 cells, however, are more reflective of the basal-like subtype of breast cancer as previously described [3]. Owing to the wide range of different tumour subcategories and levels of EGFR expression within basal-like tumours, it is significant that we can demonstrate here the effectiveness of silencing TNK2 even when the EGFR pathway is active but not hyperactivated.

## Conclusion

**Figure 6:**
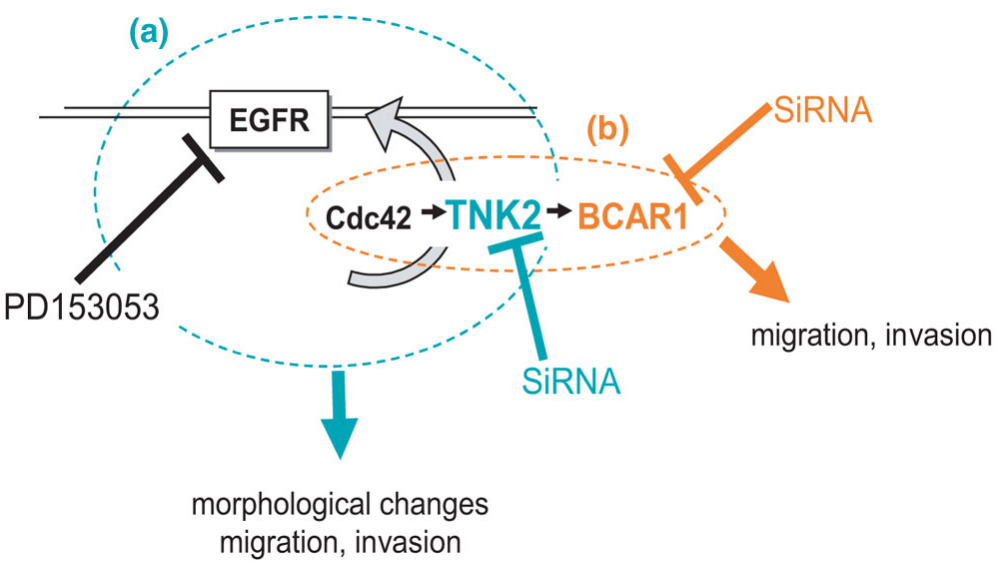
**Proposed role of TNK2 in invasion and metastasis**. **(a) **We propose that the effects of TNK2 on cell migration and the cytoskeleton are mediated by epidermal growth factor receptor (EGFR). The effects may also be mediated by breast cancer antioestrogen resistance 1 (BCAR1) downstream of TNK2, but this signal is independent from that which leads to TNK2-induced EGFR cell surface stabilisation. Previous work showed Cdc42 functions in a positive feedback loop to maintain EGFR on the cell surface, and we suggest that TNK2 functions as part of this loop. Experiments with the EGFR inhibitor PD153035 demonstrated that reduced migratory capacity results from EGFR inhibition, and that siRNA directed against TNK2 similarly inhibits migration and invasion by reduction of the number of cell surface receptors available for activation. **(b) **BCAR1 silencing, in contrast, inhibits migration and invasion downstream of Cdc42/TNK2 but does not contribute to the positive feedback effect on the EGFR.

Based on our present findings we propose that TNK2 may employ at least two distinct mechanisms to enhance breast cancer cell migration and invasion, but we note that these are not necessarily mutually exclusive. Figure 6 illustrates this proposition schematically. Furthermore, our findings suggest that TNK2 is potentially an attractive target for EGFR-dependent cancers or for cancers where the only available drugable receptor is the EGFR. There remains a need for alternative drug targets due to the unfortunate reality that the vast majority of cancers become resistant to conventional drug therapies.

The present study has demonstrated for the first time that breast cancer cell invasion can be enhanced by the ability of TNK2 to maintain EGFR cell surface expression and may provide the impetus for exploration of TNK2 as an alternative drug target for the treatment of EGFR-dependent cancers. This potential use is encouraged by the fact that the TNK2–EGFR interaction is most probably amenable to small peptide interference, as has been previously demonstrated for the Cdc42–TNK2 interaction [30,31].

## Abbreviations

BCAR1 = breast cancer antioestrogen resistance 1; BSA = bovine serum albumin; DMEM = Dulbecco's modified Eagle's medium; EGF = epidermal growth factor; EGFR = epidermal growth factor receptor; FACS = fluorescence-activated cell sorting; FBS = foetal bovine serum; N = negative control small interfering RNA; PBS = phosphate-buffered saline; RT = room temperature; siRNA = small interfering RNA.

## Competing interests

The authors declare that they have no competing interests.

## Authors' contributions

JH carried out the phase-contrast imaging, immunohistochemistry, invasion assays and FACS analysis, conceived of and designed the study, and drafted the manuscript. JR carried out the migration, proliferation and apoptosis assays. TA participated in the design and coordination of the study, and helped to draft the manuscript. All authors read and approved the final manuscript.

## Supplementary Material

Additional file 1 Adobe file comprising two figures on MCF-7 cell data. Click here for file
