# Supplementary material for: TNK2 preserves epidermal growth factor receptor expression on the cell surface and enhances migration and invasion of human breast cancer cells
Source: Breast Cancer Res. 2008 Apr 24;10(2):R36. doi: 10.1186/bcr2087 (PMC2397538; doi:10.1186/bcr2087)
Supplement: Additional file 1 — Adobe file comprising two figures on MCF-7 cell data. [file bcr2087-S1.pdf]

A

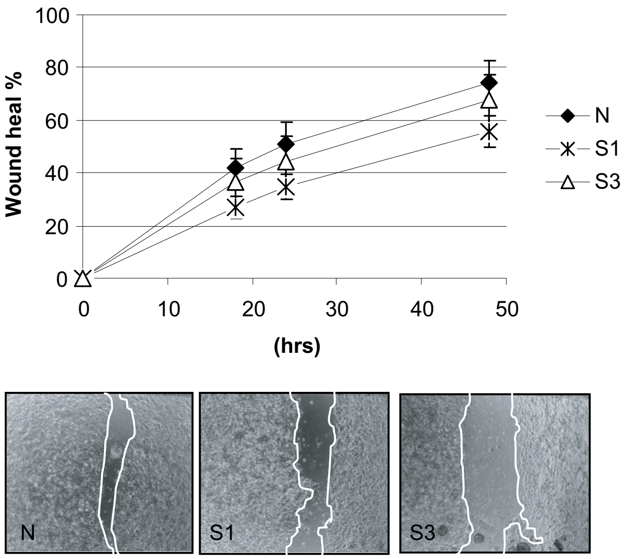

B

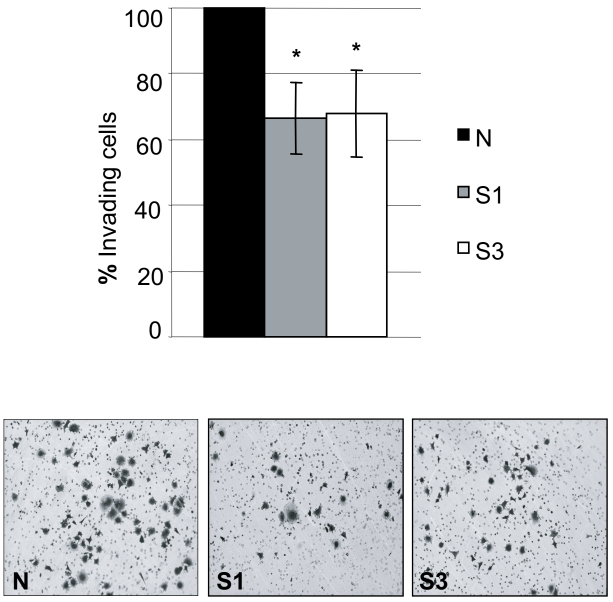

A. MCF-7 cells treated with TNK2 targeting (S1, S3) siRNA had less abilit y to migrate relative to (N) as measured by a scratch-would healing assay in serum starved cells stimulated with EGF (10ng/ml). There was a significant difference between the non-targeting SiRNA control (N) and TNK2 targeting (S1) at 48 hours, P-value = 0.0146. Underneath, representative pictures of the scratch-would assays are shown at 48 hours following wounding. **B.** An invasion assay was carried out using MCF-7 transfected with targeting (S1, S3) and non-targeting (N) SiRNA. At 48 hours post–transfection, cells were detatched, counted, added to modified boyden chambers coated with matrigel and allowed to invade over a period of 48 hours. Following this, cells which had invaded were fixed *in situ*, stained and counted. The assay was repeated a total of six times revealing an average inhibition of invasion of 33.5% (S1) and 32.7% (S3) relative to the non-targeting SiRNA control (N). There was a significant difference between the non-targeting SiRNA control (N) and TNK2 targeting (S1) and (S3) , P-value = 0.0122 and 0.038, respectively. A representative image is shown illustrating the cells which had invaded the matrigel over the course of the invasion assay.
